# Monitoring Somatic Genetic Alterations in Circulating Cell-Free DNA/RNA of Patients with “Oncogene-Addicted” Advanced Lung Adenocarcinoma: A Real-World Clinical Study

**DOI:** 10.3390/ijms23158546

**Published:** 2022-08-01

**Authors:** Laura Lupini, Roberta Roncarati, Lorenzo Belluomini, Federica Lancia, Cristian Bassi, Lucilla D’Abundo, Angelo Michilli, Paola Guerriero, Alessandra Fasano, Elisa Tiberi, Andrea Salamone, Donato Michele Cosi, Elena Saccenti, Valentina Tagliatti, Iva Maestri, Silvia Sabbioni, Stefano Volinia, Roberta Gafà, Giovanni Lanza, Antonio Frassoldati, Massimo Negrini

**Affiliations:** 1 Department of Translational Medicine, University of Ferrara, 44121 Ferrara, Italy; laura.lupini@unife.it (L.L.); roberta.roncarati@gmail.com (R.R.); bsscst@unife.it (C.B.); dbnlll@unife.it (L.D.); angelo.michilli@edu.unife.it (A.M.); paola.guerriero@unife.it (P.G.); scclne@unife.it (E.S.); iva.maestri@unife.it (I.M.); stefano.volinia@unife.it (S.V.); roberta.gafa@unife.it (R.G.); giovanni.lanza@unife.it (G.L.); 2 National Research Council, Institute of Genetics and Biomedical Research, 20089 Milan, Italy; 3 Department of Oncology, University Hospital of Ferrara, 44124 Cona, Italy; lorenzo.belluomini@univr.it (L.B.); federica.lancia@unife.it (F.L.); alessandra.fasano@unife.it (A.F.); tiberielisa1@gmail.com (E.T.); andrea.salamone@unife.it (A.S.); donatomichele.cosi@edu.unife.it (D.M.C.); 4 Laboratory for Advanced Technologies and Therapies, University of Ferrara Technopole, 44121 Ferrara, Italy; sbs@unife.it; 5 Operative Unit of Pathology, University Hospital of Ferrara, 44124 Cona, Italy; vltn.tagliatti@gmail.com; 6 Department of Life Sciences and Biotechnology, University of Ferrara, 44121 Ferrara, Italy

**Keywords:** non-small-cell lung cancer, liquid biopsy, targeted therapy

## Abstract

Liquid biopsy has advantages over tissue biopsy, but also some technical limitations that hinder its wide use in clinical applications. In this study, we aimed to evaluate the usefulness of liquid biopsy for the clinical management of patients with advanced-stage oncogene-addicted non-small-cell lung adenocarcinomas. The investigation was conducted on a series of cases—641 plasma samples from 57 patients—collected in a prospective consecutive manner, which allowed us to assess the benefits and limitations of the approach in a real-world clinical context. Thirteen samples were collected at diagnosis, and the additional samples during the periodic follow-up visits. At diagnosis, we detected mutations in ctDNA in 10 of the 13 cases (77%). During follow-up, 36 patients progressed. In this subset of patients, molecular analyses of plasma DNA/RNA at progression revealed the appearance of mutations in 29 patients (80.6%). Mutations in ctDNA/RNA were typically detected an average of 80 days earlier than disease progression assessed by RECIST or clinical evaluations. Among the cases positive for mutations, we observed 13 de novo mutations, responsible for the development of resistance to therapy. This study allowed us to highlight the advantages and disadvantages of liquid biopsy, which led to suggesting algorithms for the use of liquid biopsy analyses at diagnosis and during monitoring of therapy response.

## 1. Introduction

To reach a decision on optimal targeted molecular therapies, identification of mutations in cancer genes is needed, both at first diagnosis and at relapse or at progression. Although tissue biopsy is the first option for performing molecular investigations, the frequent lack of material needed for morphological, immunohistochemical, and molecular analyses suggests the introduction of “liquid biopsy” in clinical practice.

The presence of DNA circulating in the blood of all individuals has been known for over 70 years [1]. Plasma cell-free DNA (cfDNA) in cancer patients has been shown to contain tumor-derived DNA (ctDNA), which exhibits the same gene changes found in cancer cells. Hence, liquid biopsy has been suggested as a possible surrogate for neoplastic tissue [2,3]. Preclinical studies have provided an understanding of the importance of information derived from circulating cell-free DNA/RNA in tumor patients [4,5,6,7].

Liquid biopsy has a number of advantages over tissue biopsy. The low invasiveness and repeatability of the procedures are factors clearly in favor of liquid biopsy, allowing, for example, multiple sampling during a patient’s follow-up. From a biological point of view, by its ability to collect material released in circulation by primary tumors and metastases, liquid biopsy is more representative of tumor heterogeneity. For these reasons, liquid biopsy can be used to monitor tumor evolution over time so as to identify molecular alterations related to the development of resistance to therapy and the progression of the disease [8]. Indeed, a good concordance has been described between the mutations detected in tumors and those found in plasma [9,10]. It has also been shown in advanced lung cancer that the administration of molecularly targeted therapies based on the results of liquid biopsy investigations results in response rates comparable to those obtained following tissue investigations [10]. Therefore, clinical guidelines indicate that the use of liquid biopsy is acceptable to identify patients eligible for treatment with drugs against EGFR alterations when tissue biopsy is not available [11,12]. However, liquid biopsy also has limitations, mainly related to the low amount of ctDNA/ctRNA, a problem that can limit or even prevent the possibility of detecting cancer-associated mutations, an issue that is generally absent in the case of tissue biopsies.

In this study, we aimed to evaluate the usefulness of liquid biopsy in the clinical management of patients with advanced-stage non-small-cell lung adenocarcinoma (NSCLC), as well as candidates for or in treatment with molecularly targeted therapies. Since in NSCLC, in addition to EGFR, a number of additional gene alterations, such as mutations in ALK, BRAF, KRAS (G12C), and MET; gene fusions involving ALK, ROS1, NTRK1, NTRK2, NTRK3, and RET; and exon 14 skipping in MET, can be targeted by drugs available for clinical use, next-generation sequencing (NGS) was the main methodological approach employed in this study. The investigation was conducted on a series of cases collected in a consecutive prospective manner, which allowed us to evaluate the benefits and limitations of the approach in a real-world clinical context. The goal was to understand how this approach could be integrated into existing diagnostic and therapeutic programs: (1) at first diagnosis, compared with diagnostic investigations carried out on tissue biopsy, and (2) during patient treatment to monitor disease progression, compared with diagnostic imaging approaches.

## 2. Results

### 2.1. Patient Cohort

Plasma samples were collected from a consecutive series of 57 patients affected by advanced “oncogene-addicted” NSCL adenocarcinoma. The study was carried out on 641 plasma samples from 57 patients: 13 collected at first diagnosis, before the start of therapy, and an additional 628 plasma samples from 55 patients collected during the periodic follow-up visits; two patients (F-192 and F-253) died a few weeks after collection of the first blood sample.

This case series was made of patients enrolled progressively from July 2018 to December 2021 (Table 1). The mean duration of follow-up was 18 months (range: 2–42 months). In February 2022, 60% of the patients were alive. The patients were all followed up at the Medical Oncology Unit of the University Hospital of Ferrara. The patients underwent periodic clinical evaluation and instrumental diagnostic investigations. During each visit, peripheral blood was withdrawn to isolate plasma, which was used to purify cfDNA and cfRNA.

### 2.2. Gene Mutations in Tissue vs. Liquid Biopsy at Diagnosis

Thirteen patients enrolled in the study at the time of first diagnosis made it possible to compare the mutations identified in tissue and liquid biopsies. The results demonstrated agreement in 10 cases (77%): 7 EGFR mutations, 1 KRAS G12C mutation, 1 ALK fusion, and 1 ROS1 fusion (Table 2). In the remaining 3 cases (1 EGFR exon 19 deletion and 2 ALK fusions), no alteration was found in the liquid biopsy. It is possible that the mutant genes were present in the circulation at a level below the detection limit of the test. These results indicate that, with the exception of ALK fusions, liquid biopsy represents a good alternative to tumor tissue, if the latter is not available.

### 2.3. Liquid Biopsy to Evaluate Disease Progression

We evaluated the potential of liquid biopsies in monitoring 55 patients during therapy. Samples were collected over a period ranging from 2 to 42 months since diagnosis (18 months on average) during the periodic follow-up visits.

During the observation period, 19 patients did not exhibit disease progression according to RECIST or clinical assessment (average follow-up since first liquid biopsy: 17.0 months; range: 5–41 months). In 18 of the 19 patients (94.7%), no mutations associated with circulating DNA/RNA of tumor origin could be detected in any of the liquid biopsy samples. In only 1 case (F-372), mutations in the KRAS and PIK3CA genes were detected in the last collected plasma sample. The lack of evidence of disease progression according to RECIST in this patient is likely related to the short available follow-up (15 days) after the positive ctDNA was detected in the liquid biopsy.

**Figure 1:**
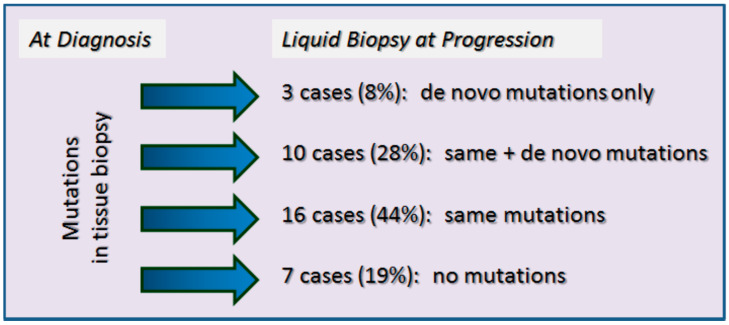
Summary of mutation detection in liquid biopsies of NSCLC cases at disease progression.

Conversely, 36 patients exhibited tumor progression (average follow-up: 18.0 months; range: 2–42 months) in accordance with the RECIST criteria or death. The molecular investigations on plasma DNA/RNA revealed the appearance and progressive increase in the circulation of tumor-associated mutations in 29 patients (80.6%). In the remaining 7 patients (19.5%), no detectable mutation was found in the liquid biopsy test at disease progression. Some of the patients experienced more than 1 event of disease progression (RECIST or clinical) during the follow-up period: Overall, 36 events were registered in the 29 patients. The alterations found in the plasma during disease progression are shown in Table 3. In 32 cases, we observed the reappearance of the same mutation present in the primary tumor, 10 of which were accompanied by additional new mutations. In 3 cases (patients F-206, F-207, and F-336), only a new mutation was detected in the absence of the original one (Figure 1).

**Figure 2:**
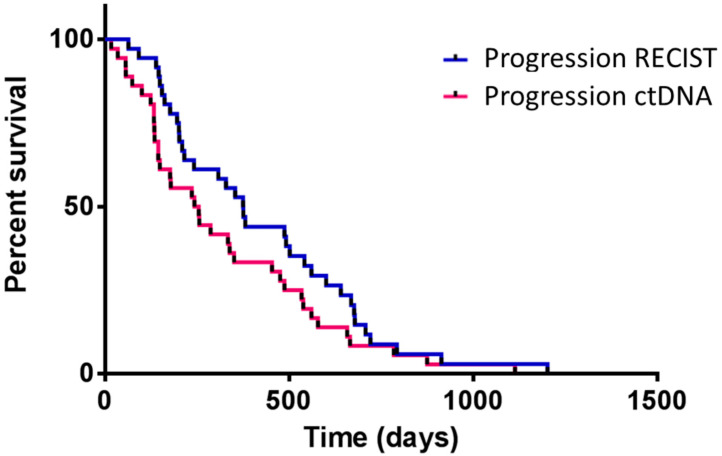
Detection of disease progression according to RECIST or by reappearance of ctDNA. X-axis represents the time (days) from 1 July 2018 (start of liquid biopsy sample collection) or the day of first diagnosis (if after 1 July 2018). Median progression “survival” was 375 days on RECIST and 249 days on ctDNA. Based on the Gehan–Breslow–Wilcoxon test, the two curves are significantly different (*p* = 0.04).

With one exception, in all cases, ctDNA alterations were detected before evidence of the progression event. The appearance of and progressive increase in the mutant allelic fraction was constantly associated with disease progression a few weeks or months later. In Table 3, it can be appreciated how the appearance of circulating mutations generally anticipated (80 days on average) the evidence of disease progression, according to RECIST. Figure 2 shows Kaplan–Meier curves of disease progression, according to RECIST or the appearance of ctDNA. These results clearly indicate that the appearance of circulating cancer-associated mutations is an early sign of disease progression.

As mentioned above, in 7 patients (about 20%) at disease progression, no mutation in ctDNA was found, indicating that a lack of gene mutations does not guarantee a nonrecurrence status of the disease in a small fraction of patients.

Not only is the detection of tumor-associated mutations in plasma relevant to monitor the response to therapy by quantitative analyses, but also it can reveal novel mutations responsible for nonresponsiveness to therapy (Table 3). In 22 cases at progression, we detected only the original mutations found in tissue at diagnosis. While these findings provide useful biomarkers of disease progression, they do not provide any indication for alternative therapeutic options other than chemo or immune therapies. In the remaining 13 cases, new mutations were detected. Such mutations were likely responsible for the development of resistance to therapy. Mechanisms of resistance to erlotinib or gefitinib included EGFR T790M in 5 cases and KRAS G12C in 1 case. Mechanisms of resistance to osimertinib included EGFR C797S in 1 patient and BRAF V600E in another patient. Oddly, EGFR T790M was also found in 5 cases on osimertinib therapy.

In summary, while the quantification of circulating mutant allelic fraction can function as a biomarker that inversely correlates with response to therapy in 80.6% (29/36) of the patients, in approximately one-third of progression events (13/35), liquid biopsy analyses led to the identification of novel mutations that were responsible for therapy resistance, which could eventually provide indications for alternative molecularly targeted therapies. This aspect represents an added value of liquid biopsy over other approaches employed for monitoring patients during follow-up.

### 2.4. Some Cases Exemplifying Patients’ Follow-Up

Some cases exemplify how the quantitative and qualitative changes of circulating mutations correlated with clinical variations (Supplementary Figures S1–S4). Supplementary Figure S1 shows an example of such a concept. The occurrence of the EGFR T790M mutation in disease progression during gefitinib therapy could be counteracted with osimertinib. The graph shows a quantitative inverse correlation between ctDNA and the response to therapy. Similarly, the occurrence of the EGFR C797S mutation, case F-238 (Supplementary Figure S2), was instead associated with resistance to osimertinib [13,14]. The appearance of BRAF V600E mutations, case F-249 (Supplementary Figure S3), was also associated with resistance to osimertinib. While, as of today, the use of dabrafenib plus trametinib is indicated for this condition, no targeted therapeutic approach was clinically available at the time when the mutation was discovered in the patient. The fourth case was associated with a ROS1 fusion (Supplementary Figure S4): an increase in the EZR–ROS1 fusion was detected upon liquid biopsy approximately 3 weeks after indication of a partial response and 1 month before evidence of disease progression. In summary, the quantitative increase in ctDNA/ctRNA mutations in plasma was constantly early evidence of the loss of responsiveness to therapy. Furthermore, the appearance of novel mutations could eventually provide indications for an alternative therapy. For example, besides the use of osimertinib in cases of development of EGFR T790M in the presence of gefitinib or erlotinib (cases F-193, F-203, and F-209), the appearance of KRAS G12C (case F-207) could be counteracted by sotorasib, or the appearance of BRAF V600E (case F-249) could be counteracted by dabrafenib plus trametinib.

### 2.5. Clinical Algorithms That Incorporate Liquid Biopsy in Diagnostic and Therapeutic Programs

**Figure 3:**
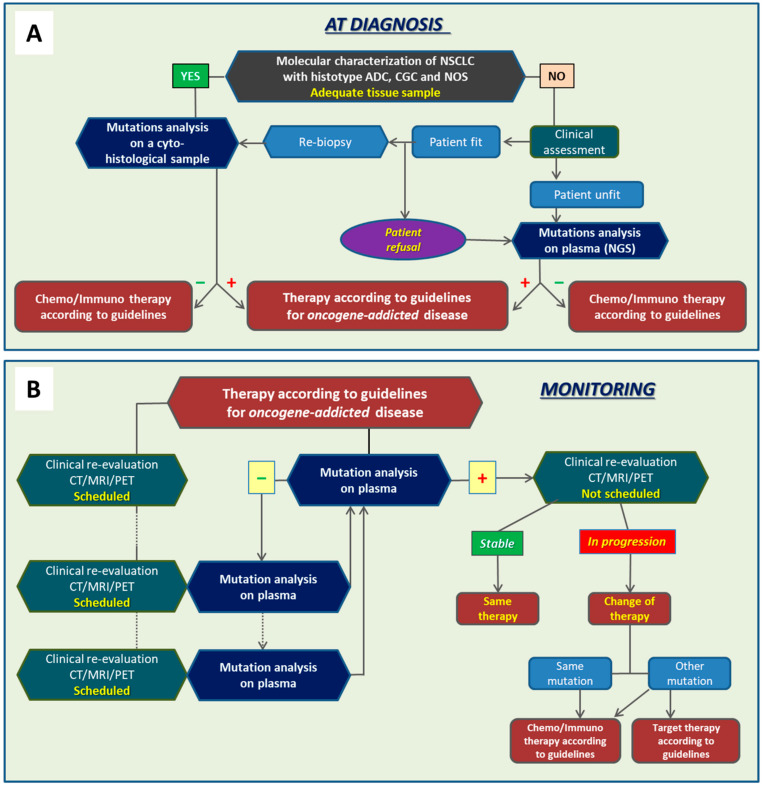
Clinical algorithms for the use of liquid biopsy at diagnosis (**A**) and during follow-up (**B**).

In accordance with our results, we propose algorithms that incorporate liquid biopsy approaches at diagnosis and during monitoring of the therapy response of patients with advanced-stage oncogene-addicted NSCLC (Figure 3).

At diagnosis, molecular investigations should be performed on a tumor tissue sample using an NGS approach. Only when the material is qualitatively or quantitatively insufficient should liquid biopsy be considered (Figure 3A). In fact, liquid biopsy cannot reach 100% concordance with molecular results from tissue specimens. This is particularly true for gene fusions.

Liquid biopsy can instead find a wider application during the monitoring of therapy efficacy. In fact, considering the need to collect multiple points to monitor this phase in real time, liquid biopsy represents an optimal approach to investigate disease biomarkers. Liquid biopsy samples can be collected during routine follow-up visits and at the time of imaging investigations. If the results of the test are negative, the routine is repeated at every visit. If positive, clinical and imaging evaluations should be intensified until evidence of disease recurrence occurs and a change in therapy becomes possible (Figure 3B). The therapeutic options depend on the mutations detected in ctDNA/ctRNA. For patients who progress but do not show any gene mutation in ctDNA, they keep receiving follow-ups using standard monitoring approaches (Figure 3B).

The optimal method for performing analyses on ctDNA/ctRNA remains to be defined. At diagnosis, NGS is unquestionably the optimal approach. During follow-up monitoring, NGS is also the preferable method before knowledge of ctDNA mutations. In fact, analysis of a wide panel of genes allows us to uncover all potential new situations that should occur at progression. Once a gene biomarker of progression is discovered, the use of quantitative PCR (ddPCR or other quantitative PCR methods) would instead be preferable in terms of speed and costs.

## 3. Discussion

This study evaluated the usefulness of liquid biopsy in a real-world clinical setting on a group of consecutive patients with advanced-stage lung adenocarcinoma undergoing treatment with molecularly targeted therapies. The goal was to define how liquid biopsy could be integrated into currently active diagnostic and therapeutic programs, both at diagnosis and at disease progression.

In regard to the use of liquid biopsy at diagnosis, our results suggested that, to a large extent, it can be employed in the diagnostic assessment of advanced stages of disease, but it cannot fully replace analyses performed on tissue biopsies. Published studies comparing the two types of investigations are in agreement with this conclusion, as they reported an overall agreement in approximately 90% of the cases [15,16]. In support of the clinical value of liquid biopsy in advanced stages is the finding that the response rates of patients with NSCLC positive for EGFR–T790M in plasma exhibited outcomes equivalent to patients who were classified by a tissue-based assay [17,18]. In the present study, an adverse issue that surfaced was the detection of gene fusions in ctRNA [19]. Albeit we could usually detect ALK and ROS1 gene fusions in ctRNA, two of the three undetected gene alterations at diagnosis were actually ALK fusions, implicating that the detection of ALK fusions in RNA from plasma samples might be challenging. Hence, the availability of tissue material appears to be particularly important for detecting ALK, ROS1, and NTRK alterations by IHC expression analyses and gene fusions by NGS.

It remains to be evaluated whether the detection of ctDNA/ctRNA can be applied to early stages of NSCLC. In fact, ctDNA is approximately 100-fold higher in stage IV compared with stage I tumors [20]. Although the sensitivity of technology is significantly improved in recent years, liquid biopsy still cannot be applied at present to the search of ctDNA mutations in these early stages. This aspect may become an issue, considering that osimertinib is now indicated as an adjuvant therapy in the early stages of NSCLC [21,22].

Thus, the indication that emerges from our study, as well as from published studies in advanced NSCLC, is that liquid biopsy cannot fully replace analyses performed on tumor tissues. Molecular investigations at diagnosis should be carried out on tissue biopsy whenever possible, with NGS being the preferred methodological approach. Only if material is quantitatively or qualitatively insufficient does it seem indicated to proceed with NGS analysis using liquid biopsy (Figure 3A).

Regarding liquid biopsy for assessing response to therapy, it has been reported that longitudinal analysis of ctDNA allows quantitative tracking of tumor burden and treatment response in various human cancers [2,9,23,24,25,26]. It has also been shown that the quantitative dynamic changes of ctDNA in plasma correlated with tumor size, stage, and differential prognosis [9,27,28,29]. Our results indicate that the appearance or re-emergence of mutations in ctDNA during therapy is an early sign of resistance to therapy. In all of the cases in our study, the re-emergence of mutations in ctDNA was followed within a few weeks or months by disease progression, according to RECIST or clinical evidence. In the absence of approved clinical protocols that allow therapy changes based on ctDNA evidence only, the patient should be more frequently monitored using standard approaches until evidence of disease progression occurs (Figure 3B). The therapeutic options depend on the mutations detected in ctDNA/ctRNA. In cases where the same mutations of primary tumor or “nonactionable” mutations are found, the use of chemotherapy or immunotherapy according to current guidelines is the most likely indication. Instead, if a de novo “actionable” mutation emerges, the use of an appropriate molecularly targeted drug would be preferable, and its use should be evaluated according to guidelines or other clinically available standards (Figure 3B). Finding new “actionable” mutations represents a unique value of liquid biopsy during the monitoring of therapy efficacy. Unfortunately, cases that can take advantage of molecularly targeted therapies as a second line of therapy are currently a minority. Overcoming osimertinib resistance is an area still under investigation. It has been reported that a combination of anti-EGFR drugs with brigatinib may be effective in some cases of resistance to osimertinib [30,31,32,33,34,35], and fourth-generation anti-EGFR drugs are being studied, but not yet approved for clinical use [36,37]. Far fewer therapeutic options have been developed for other targets. Drugs have been developed for patients resistant to first- and second-line therapy for ALK rearranged tumors, such as alectinib and lorlatinib, or for ROS1 rearranged tumors, such as entrectinib and larotrectinib. However, the trend to use the most recent and effective drug as the first line reduces the options for the treatment of resistance to the latest generations of targeted drugs.

In case of occurrence of a mutation in a different gene, such as a KRAS G12C or BRAF V600E mutation (as in the case we observed), MET exon 14 skipping mutation, or RET rearrangement, specific drugs targeting the new molecular alteration can be used. Less options are available for other mutations, such as HER2 and PIK3CA, even if an agnostic approach might be considered.

Our results provide evidence that liquid biopsy can detect disease progression approximately 80 days on average earlier than imaging approaches. Other studies have also reported similar findings. Taus and Sorensen demonstrated that the detection of EGFR mutations in TKI-treated patients was able to predict radiological response and disease progression with an anticipation of more than 1 month [38,39]. In another study, plasma investigations on 25 patients receiving osimertinib identified mutations in EGFR (C797S and Q791P) and other resistance-associated genes (KRAS, BRAF, and PIK3CA) several months earlier than the evidence of clinical progression [40]. In bladder cancer, a median anticipation time of 96 days has been reported for liquid biopsy over radiographic imaging [41].

In a rapidly evolving disease, such as lung cancer, identifying the onset of recurrence as early as possible may potentially be important. However, in an advanced clinical setting, the question concerning the potential usefulness of starting a new line of therapy a few months earlier than the current standards remains an unanswered question and deserves specifically designed trials. In breast cancer, for example, the PADA1 study addressed this specific question using ESR1 as a candidate biomarker [42]. Patients in which an ESR1 mutation was detected in plasma ctDNA were randomized to continue the same therapy or to change treatment. Early change resulted in a statistically better outcome, giving an indirect confirmation of the potential utility of the early change strategy. However, other aspects related to, for example, the type of assay and to the acceptable threshold of ctDNA alteration useful for switching therapy await further analyses.

Our results showed that tumor progression can also occur without any evidence of re-emergence of mutations in ctDNA in about one-fifth of events of disease progression (7/36). The reason is not known. Besides the possibility that a mutant allele was below the detection limit of the test, we cannot exclude that mutations occurred in genes not present in the panel employed in the present study, or that copy number variations, which are difficult to detect in ctDNA, were responsible for the recurrence. These cases are to be followed up according to standard clinical practice, which includes periodic scheduled visits and diagnostic investigations based on CT, MRI, or PET (Figure 3B). Chemotherapy- or immunotherapy-based approaches remain an available alternative to target therapy.

While in 2018, an ASCO panel of experts concluded that “there is insufficient evidence of clinical validity and utility for the majority of ctDNA assays in advanced cancer” [43], more recent recommendations have changed this view. The current National Comprehensive Cancer Network Clinical Practice Guidelines for molecular and biomarker analysis of NSCLC indicate that ctDNA can be employed in medically unfit patients (www.nccn.org/guidelines/guidelines-detail?category=1&id=1450 accessed on 1 February 2022), and the International Association for the Study of Lung Cancer (www.iaslc.org/iaslc-news/press-release/iaslc-issues-consensus-updated-report-liquid-biopsies accessed on 1 February 2022), as well as the Italian AIOM-SIAPEC-IAP-SIBIOC-SIF Working Group (https://www.siapec.it/2020/07/01/raccomandazioni-2020-per-lesecuzione-di-test-molecolari-su-biopsia-liquida-in-oncologia/, accessed on 1 February 2022), concluded that liquid biopsy is complementary to tissue-based analysis; however, it is an acceptable approach at diagnosis and for monitoring the efficacy of targeted therapies. Our conclusions are in line with these latter recommendations.

## 4. Materials and Methods

*Study design and patients.* This single-center and prospective study was conducted at the University Hospital of Ferrara from July 2018 to December 2021. Fifty-seven patients were prospectively enrolled in the study (Table 1). All patients were diagnosed as having stage III or IV lung adenocarcinoma carrying actionable mutations in the primary tumor. They were all on treatment with targeted therapy according to clinical guidelines. The study was approved by the local ethics committee. Before enrollment, each patient was informed in a detailed and understandable manner about the study and signed an informed consent. For the study purposes, a unique anonymized code was assigned to each patient. During treatment, the patients were periodically subjected to clinical evaluations to monitor therapy efficacy and the development of local or distant recurrences, according to the “response evaluation criteria in solid tumors” (RECIST). Blood samples for research purposes were obtained during each visit. Clinical, imaging, pathology, and molecular data of each patient were collected in a longitudinal prospective manner and were related to one another at the end of the study.

*Nucleic acid purification and quantification.* Peripheral blood samples (10 mL) were collected in two EDTA vacutainer tubes. To obtain plasma samples, blood was centrifuged at 1000 rcf for 10 min. Aliquots of plasma were stored at −80 °C until thawed just before nucleic acid purification. DNA and RNA were purified from plasma using the automated extractor Maxwell RSC and the Maxwell^®^ RSC miRNA Plasma and Serum Kit (Promega), according to the manufacturer’s indications. Isolated nucleic acids were quantified by Qubit^®^ 2.0 fluorimeter (Thermo Fisher, Waltham, MA, USA), according to the manufacturer’s procedures.

*Droplet digital PCR (ddPCR).* The ddPCR assays were performed on the QX200 Droplet Digital PCR System (Bio-Rad Laboratories, Contra Costa, CA, USA). To quantify EGFR mutations, the Bio-Rad allele-specific Taqman assays EGFR p.T790M c.2369C> T (probe conjugated to FAM fluorophore) and EGFR WT for p.T790M c.2369C> T (probe conjugated to HEX fluorophore) were used to detect and quantify the EGFR T790M mutation and its wild-type counterpart. EGFR p.L858R c.2573T> G (probe conjugated to FAM fluorophore) and EGFR WT for p.L858R c.2573T> G (probe conjugated to HEX fluorophore) were used to detect and quantify the variant L858R and its corresponding wild-type counterpart. The procedures were in accordance with the manufacturer’s indications. The results were recorded using QuantaSoft (Bio-Rad) software. The mutation frequency was calculated as the ratio of the number of positive droplets for the mutant allele (FAM-positive) to the total number of positive droplets (FAM + HEX positive droplets). To quantify the fusion between EZR exon 10 and exon 34 of ROS1, the EZR–ROS1 assay Hs04396902_E10;R34 (Thermo Fisher) was employed. Before PCR amplification, a retrotranscription was performed on cfRNA (30 ng) using a SuperScript™ VILO™ cDNA Synthesis Kit (Thermo Fisher).

*Next-generation sequencing.* The NGS investigations were performed with the Ion GeneStudio^TM^ S5 System (Thermo Fisher) technological platform. The sample libraries were prepared using an Oncomine Lung Cell-Free Total Nucleic Acid Research Assay Kit (Thermo Fisher). This is an amplicon-based assay that allows the detection of gene mutations in ALK, BRAF, EGFR, ERBB2, KRAS, MAP2K1, MET, NRAS, PIK3CA, ROS1, and TP53; gene fusions involving the ALK, RET, and ROS1 genes; and MET exon 14 skipping. The kit, which includes UMIs (“unique molecular identifiers” or “tags”), allows the identification of mutations down to 0.1% of the variant allelic frequency. Libraries were prepared according to the manufacturer’s instructions, starting from 20 ng cfDNA/cfRNA. Sequencing was performed with the Ion GeneStudio^TM^ S5 System (Thermo Fisher) sequencer on an Ion 540 Chip, which generates 60–80 million reads (10–15 Gb).

Data analysis was performed using the Torrent Suite, version 5.10.1. For each target region of each sample, the “total depth” (the total number of reads obtained for each region) and the “molecular depth” (the number of reads with different UMIs) were assessed. A molecular depth of at least 2000 reads was required to reach a 0.1% limit of detection at each region. On average, we obtained between 4000 and 5000 reads of molecular depth.

The data preprocessed by the Torrent Suite were then uploaded to the Ion Reporter software, version 5.10.5 (Thermo Fisher), to report all of the identified gene alterations in each sample. For each mutation, the software reports the type of mutation, its genomic localization, its allelic frequency, the detection limit at that specific region, the total and molecular depth of the region, and the pathogenicity of the variant predicted by bioinformatics algorithms (e.g., PolyPhen 2 and SIFT). All of the pathogenic or potentially pathogenic sequence variants were visually verified by uploading the reads to the Integrative Genomics Viewer (IGV) program [44,45].

## Figures and Tables

**Table 1:** Patient cohort.

Features		Number
**Patients**	Male	18
	Female	39
**Alive/deceased**		34/23
**Follow-up (from first liquid biopsy)**	Average (range) months	18 (2.42)
**Age at diagnosis**	Average (range)	67 (39–88)
**Smoking habits**	Smokers	9
	Ex-smokers	22
	Nonsmokers	26
**Histology**	Adenocarcinoma	52
	Mucinous adenocarcinoma	2
	Poorly differentiated carcinoma	2
	Giant cell carcinoma	1
**Stage (TNM)**	III	3
	IV	54
**Metastasis sites**	Lung	35
	Lymph nodes	45
	Bone	22
	Liver	10
	Pleura	12
	Brain	17
	Spleen	2
	Adrenal glands	4
	Peritoneum	3
	Soft tissue	2
	Pericardium	1
	Kidney	1
**Gene alteration in the primary tumor**	EGFR exon 19 del	22
	EGFR L858R	16
	EGFR G719A, G719S	3
	ALK *	7
	ROS1 *	3
	KRAS G12C	5
	BRAF V600E	1
	EGFR E709K	1
	EGFR T790M	1
	EGFR L861Q	1
**TKIs in the first line**	Gefitinib	16
	Osimertinib	23
	Crizotinib	7
	Alectinib	4
	Erlotinib	3
	Sotorasib	3
	Dabrafenib–trametinib	1

* ALK and ROS1 were analyzed using IHC on primary tumor tissue.

**Table 2:** Liquid biopsy at diagnosis.

Patient	Gene Alterations in Primary Tumors at Diagnosis	Gene Alterations in Liquid Biopsy at Diagnosis
F-192	EML4–ALK fusion	EML4(13)–ALK(20)
F-204	EML4-ALK fusion	None
F-213	EGFR L858R	EGFR L858R
F-221	ROS *	EZR(10)–ROS1(34)
F-225	EGFR exon 19 del	EGFR exon 19 del
F-238	EGFR exon 19 del	EGFR exon 19 del
F-250	EGFR L858R	EGFR L858R
F-253	EGFR L858R	EGFR L858R
F-260	EGFR G719A	EGFR G719A
F-297	EGFR del exon 19	EGFR del exon 19
F-307	EGFR del exon 19	None
F-324	EML4–ALK fusion	None
F-372	KRAS G12C	KRAS G12C

* ROS1 was analyzed using only IHC on tissue.

**Table 3:** Mutations at patients’ progression detected by liquid biopsy.

Patient	Mutations in the Primary Tumor	Therapy	Mutations at Disease Progression	Anticipation on RECIST ^1^ (days)
F-182	EGFR p.L747-S751del	Gefitinib	EGFR p.L747-S751del	47
F-183	EGFR p.L858R	Gefitinib	EGFR p.L858R	88
F-188	EGFR p.E746_A750del	CT	EGFR p.E746_A750del	98
F-188	EGFR p.E746_A750del	Osimertinib	EGFR p.E746_A750del	247
F-193	EGFR p.E746-A750del	Gefitinib	EGFR p.E746-A750del + EGFR p.T790M	56
F-194	EGFR p.G719A	Gefitinib	EGFR p.G719A	82
F-194	EGFR p.G719A	Osimertinib	EGFR p.G719A	13
F-196	EGFR p.T790M; p.L858R	Osimertinib	EGFR p.T790M + EGFR p.L858R	97
F-200	EGFR p.L858R	Gefitinib	EGFR p.L858R	8
F-201	EGFR p.L747_A750delinsP	Osimertinib	EGFR p.L747_A750delinsP	0
F-203	EGFR p.L858R	Gefitinib	EGFR p.L858R + EGFR p.T790M	175
F-203	EGFR p.T790M	Osimertinib	EGFR p.L858R + EGFR p.T790M	197
F-205	EGFR p.T790M	Osimertinib	EGFR p.E746_A750del + EGFR p.T790M	203
F-206	EGFR p.E746-A750del	Erlotinib	EGFR p.E746_A750del	28
F-206	EGFR p.T790M	Osimertinib	EGFR p.T790M	94
F-206	EGFR p.T790M	Beva-atezo	EGFR p.E746_A750del + EGFR p.T790M + EGFR p.C797S	25
F-207	EGFR p.E746-A750del	Gefitinib	KRAS p.G12C	36
F-209	EGFR p.L858R	Gefitinib	EGFR p.L858R + EGFR p.T790M	132
F-209	EGFR p.L858R	Osimertinib	EGFR p.L858R + EGFR p.T790M	290
F-213	EGFR p.L858R	Gefitinib	EGFR p.L858R + EGFR p.T790M	93
F-221	ROS1	Crizotinib	EZR(10)-ROS1(34)	42
F-228	EGFR p.E746-A750del	Gefitinib	EGFR p.E746-A750del	0
F-238	EGFR p.E746-A750del	Osimertinib	EGFR p.E746-A750del + EGFR p.C797S	50
F-249	EGFR p.L858R	Osimertinib	EGFR p.L858R + BRAF V600E	49
F-250	EGFR p.L858R	Osimertinib	EGFR p.L858R	39
F-260	EGFR p.G719A	Osimertinib	EGFR p.G719A	55
F-296	EGFR p.L858R	Osimertinib	EGFR p.L858R	130
F-318	EGFR p.L858R	Osimertinib	EGFR p.L858R	67
F-336	EGFR p.G719S; p.E709K	Osimertinib	KRAS p.G12D	67
F-349	EGFR p.E746-A750del	Osimertinib	EGFR p.L747_A750del	22
F-353	EGFR p.L861Q	Osimertinib	EGFR p.L861Q	−26
F-354	EGFR p.E746-A750del	Osimertinib	EGFR p.E746_A750del	88
F-403	KRAS p.G12C	Sotorasib	KRAS p.G12C	16
F-409	KRAS p.G12C	Sotorasib	KRAS p.G12C	100
F-436	EGFR p.E746-A750del + EGFR p.T790M	Osimertinib	EGFR p.T790M	72

^1^ RECIST = Response evaluation criteria in solid tumors.

## Data Availability

Not applicable.

## Supplementary Materials

The following supporting information can be downloaded at: https://www.mdpi.com/article/10.3390/ijms23158546/s1.
