# Supplementary material for: Monitoring Somatic Genetic Alterations in Circulating Cell-Free DNA/RNA of Patients with “Oncogene-Addicted” Advanced Lung Adenocarcinoma: A Real-World Clinical Study
Source: Int J Mol Sci. 2022 Aug 1;23(15):8546. doi: 10.3390/ijms23158546 (PMC9369384; doi:10.3390/ijms23158546)
Supplement: Supplementary file 1 [file ijms-23-08546-s001.zip › ijms-1808880-supplementary.pdf]

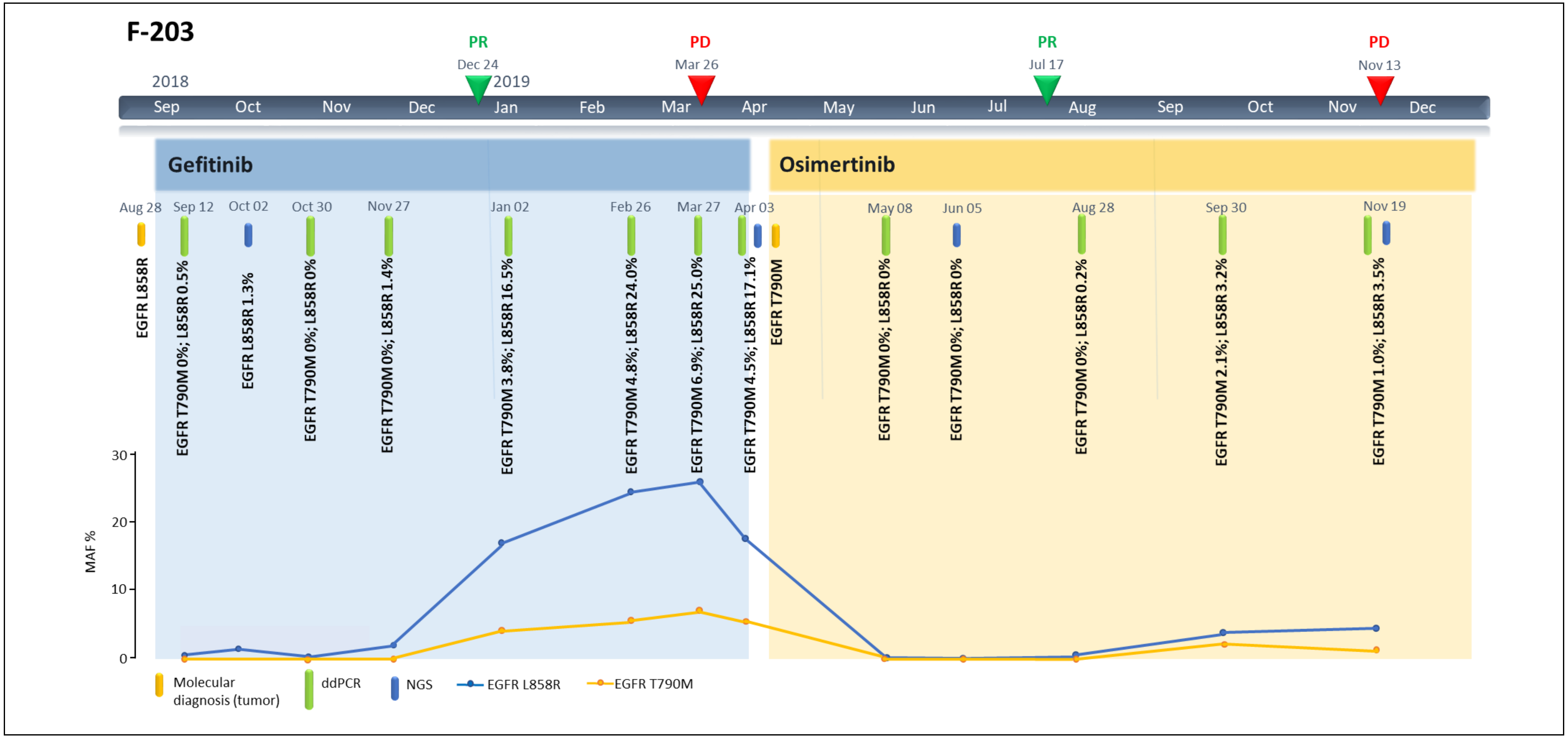

Figure S1. ***Timeline of Patient F-203.*** The timeline is from September 2018 to December 2019 (upper bar). The figure shows key dates related to patient's follow-up. Pharmacological treatments with gefitinib and osimertinib are shown by colored areas. The collection dates of liquid biopsy samples are shown by green bars (ddPCR analysis) or blue bars (NGS analysis) at the top. Orange bars indicate the molecular analyses performed on tissues biopsies. RECIST assessments are shown by green (PR, partial response) or red (PD, progression disease) triangles. The orange and blue lines at the bottom represent the L858R and T790M mutation frequencies (MAF%, mutant allele frequency) measured in liquid biopsy samples at the different time points. After evidence of PD on November 2019, therapy was shifted to chemoimmunotherapy. Patient died on July 2020.

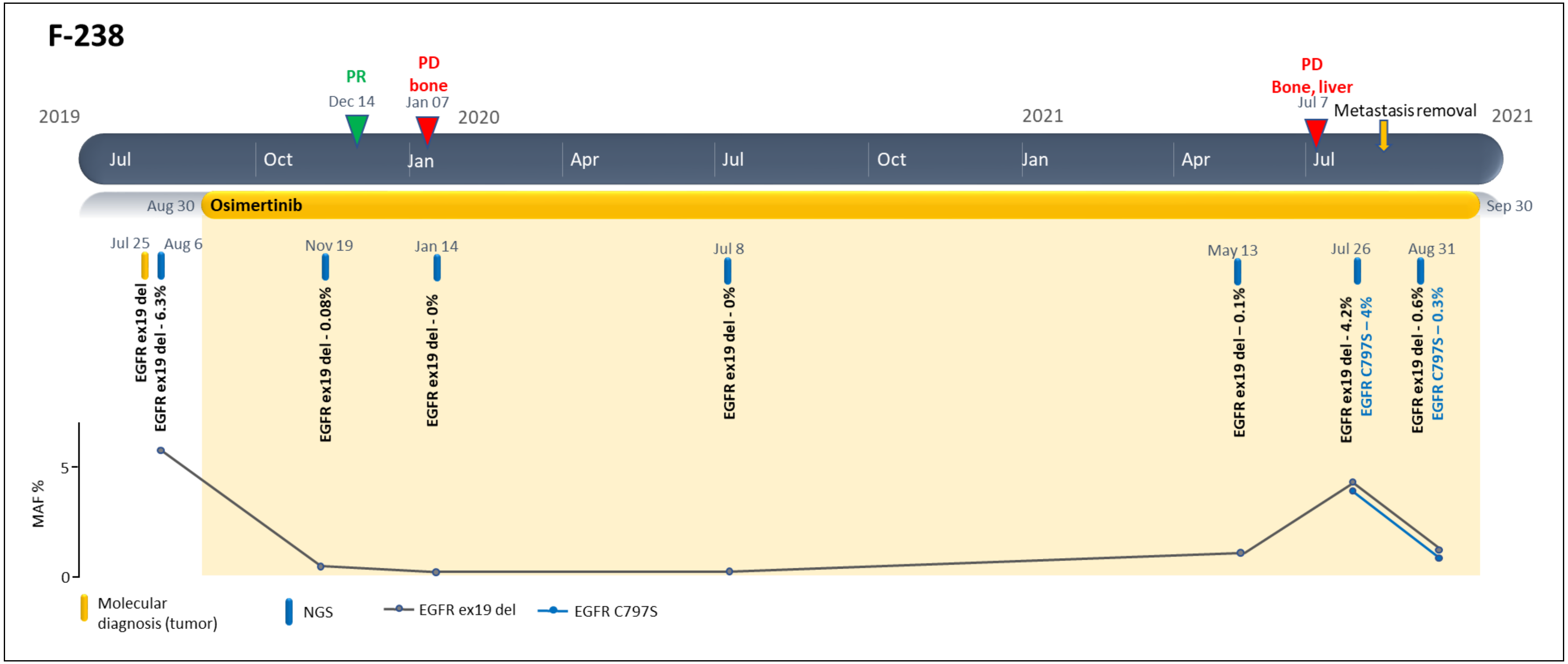

Figure S2. ***Timeline of Patient F-238***. The timeline is from July 2019 to September 2021 (upper bar). Osimertinib treatment is shown by the yellow area. The collection dates of liquid biopsy samples analyzed by NGS are shown by blue bars. Orange bar indicates the molecular analysis performed on tissue biopsy. RECIST assessments are shown by green (PR, partial response) or red (PD, progression disease) triangles. The gray and blue lines at the bottom represent the EGFR exon 19 deletion and C797S mutation frequencies (MAF%, mutant allele frequency) measured in liquid biopsy samples at the different time points. On July 2021, the patient underwent surgery to remove the metastasis, resulting in a reduction in the plasma frequencies of both EGFR alterations. Patient is alive.

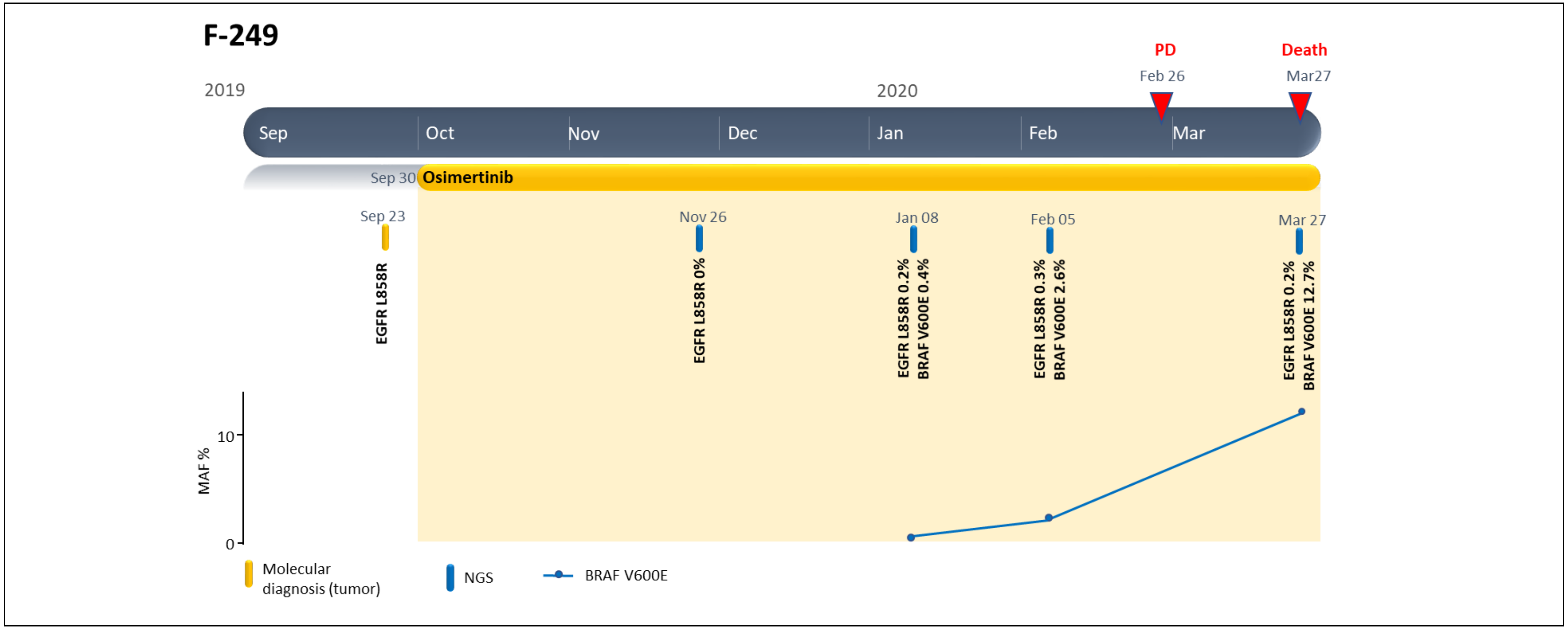

Figure S3. ***Timeline of Patient F-249.*** The figure shows F-249 patient follow-up, from September 2019 to March 2020 (upper bar). Osimertinib treatment is displayed by the yellow area. The blue bars at the top represent the collection dates of liquid biopsy samples analyzed by NGS, with EGFR L858R and BRAF V600E mutation frequencies. Orange bar indicates the molecular analysis performed on tissue biopsy. Red triangle at the top displays progression disease (PD) according to RECIST, identified in the patient through CT imaging, or death. The blue line at the bottom represent the BRAF V600E mutation frequencies (mutant allele frequency - MAF%) measured in the different liquid biopsy samples, by NGS. Patient died at the end of March 2020.

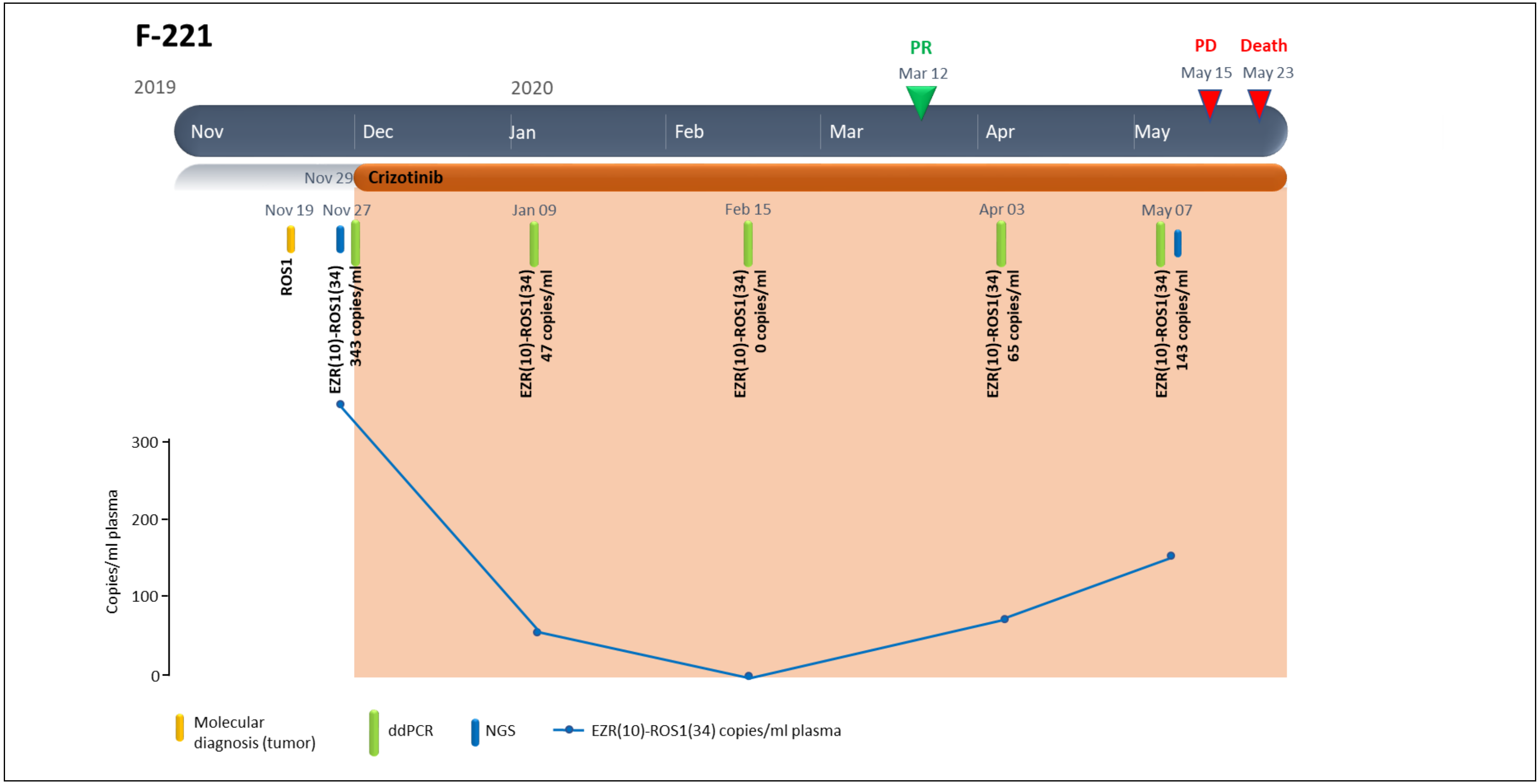

Figure S4. ***Timeline of Patient F-221.*** The figure shows F-221 patient follow-up, from November 2019 to May 2020. This patient was treated with crizotinib, as indicated by brown bar. The collection dates of liquid biopsy samples are shown by green bars (ddPCR analysis) or blue bars (NGS analysis) at the top. Orange bar indicates the molecular analysis performed on tissue biopsy. RECIST assessments are shown by green (PR, partial response) or red (PD, progression disease or death) triangles. The blue line at the bottom represents the EZR(10)-ROS1(34) fusion copies/ml plasma measured in the different liquid biopsy samples, by ddPCR. Patient died on May 2020.
